# Myasthenia Gravis-Like Syndrome Presenting as a Component of the Paraneoplastic Syndrome of Lung Adenocarcinoma in a Nonsmoker

**DOI:** 10.1155/2014/703828

**Published:** 2014-07-23

**Authors:** Sahar Eivaz-Mohammadi, Fernando Gonzalez-Ibarra, Hesam Hekmatjou, Rao Mikkilineni, Amit Patel, Amer K. Syed

**Affiliations:** ^1^Department of Internal Medicine, Jersey City Medical Center, Jersey City, NJ 07302, USA; ^2^Department of Pulmonary and Critical Care, Jersey City Medical Center, Jersey City, NJ 07302, USA; ^3^Department of Hematology and Oncology, Jersey City Medical Center, Jersey City, NJ 07302, USA; ^4^Laureate National Institute of Medicine, Program Director Internal Medicine, Jersey City Medical Center, Jersey City, NJ 07302, USA

## Abstract

Adenocarcinoma of the lung is the most common form of lung cancer in nonsmokers. It is commonly seen in the periphery of the lungs. Myasthenia gravis is generally associated with mediastinal malignancies and rarely associated with adenocarcinoma of the lung. We present a case of a 38-year-old male nonsmoker with rapidly progressive adenocarcinoma of the lung associated with myasthenia gravis, a patient whom expired within 27 days of hospital admission and diagnosis.

## 1. Introduction

Myasthenia gravis is an autoimmune neuromuscular disease caused by autoantibodies to postsynaptic acetylcholine receptors, blocking their attachment at the postsynaptic junction. This results in a lack of the excitatory effects of acetylcholine at the postsynaptic nicotinic receptors. It is most commonly found in association with autoimmune diseases including lupus erythematosus, rheumatoid arthritis, diabetes mellitus type 1, and hashimoto's thyroiditis, grave's disease, and thymic diseases such as thymoma. We present a case of a young nonsmoker diagnosed with myasthenia gravis via electromyography-nerve conduction study as a paraneoplastic manifestation of the lung adenocarcinoma.

## 2. Case Presentation

A 38-year-old male recent immigrant from the Dominican Republic presents to the emergency department with chief complaint of hemoptysis of two-week duration. The hemoptysis was associated with an increased shortness of breath and dry cough which began two months prior to admission and had progressively worsened. The patient further described right-sided chest pain, intermittent, 5/10, sharp, gnawing/dragging in nature radiating to the shoulders especially upon recumbency and relieved with sitting upright or leaning forward. He further describes neck pain and fullness, as well as progressive heaviness of the arms bilaterally with decreased strength and intermittent lack of ability to grasp objects. The patient had no significant past medical history and had never smoked in the past and no significant occupational/environmental exposures.

Further review of the patients past medical history revealed history of a doctor's visit 18 months prior to admission for similar complaints in the Dominican Republic based on reports provided by the patient; computed tomography of the chest along with “neurologic testing” were completed at that time. Documentation provided at this time by the patient of CT chest showed a right superior lobe pulmonary nodule measuring 1.8 × 1.5 cm. The patient, however, was discharged with a diagnosis of bronchitis and was treated with a course of antibiotics, and patient was lost to follow up.

Physical examination revealed tachycardia, ruddy discoloration of the face, and neck and upper chest with marked fullness noted around the neck extending from the superior aspect of the chest to the angle of the jaw. Examination revealed diminished ability and difficulty to sustain upward gaze for greater than 20 seconds was further noted. Snellen's card exam revealed visual acuity of 20/30 on the right and 20/25 on the left with subjective diplopia reported during the examination as well as during testing for finger to nose agnosia. Diminishing speed and increased difficulty to complete testing of rapid alternating movements were observed. Inability to maintain a sustained hold against gravity of the upper extremities level with the shoulders while holding the arm in an outstretched position was noted on the right greater than the left. Repeat examination following a period of rest produced similar results. No air-entry was heard at the right lung base. The physical examination was otherwise unremarkable and the patient was hemodynamically stable. A complete blood count and complete metabolic panel were also ordered and were significant for a large leukocytosis (WBC: 19.1, PMN: 75 & band: 2); rest of the laboratory tests were unremarkable (Table 1).

**Figure 1:**
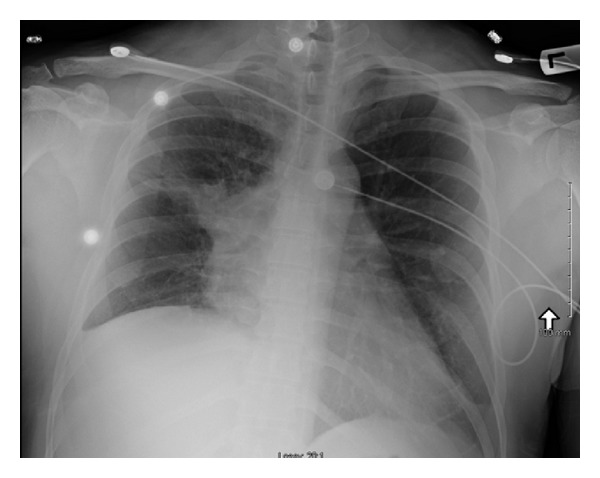
Chest X-ray showing a large right hilar mass extending to the right upper lobe with associated fine loss in the right lung. There is a right pleural effusion seen as well.

**Figure 2:**
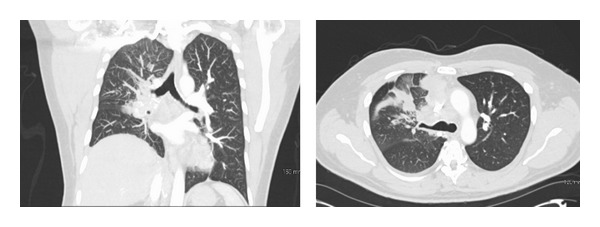
CT angiogram chest showing a large right mediastinal mass (8.8 × 6.2 × 9.6 cm), consistent with malignancy involving the right cardiac atrium with occlusion of the right main pulmonary artery which is either compressed or invaded by the mass, and nonopacification of right pulmonary arteries. No left pulmonary embolus is seen. Metastatic adenopathy in the mediastinum and probably right hilum. There are enlarged mediastinal lymph nodes, measuring up to 2.6 × 2.2 cm in the precarinal region and 2.2 × 1.9 cm in the anterior superior mediastinum.

**Figure 3:**
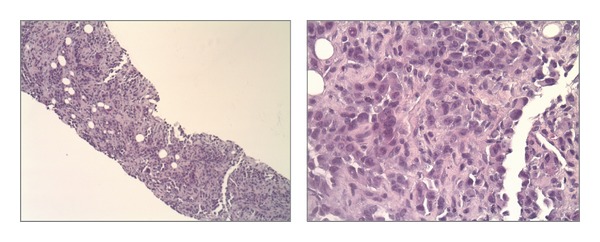
H&E stain of mass.

CT chest was ordered and revealed a large right mediastinal mass measuring 8.8 × 6.2 × 9.6 cm (Figures 1 and 2), consistent with malignancy, involving the right cardiac atrium with occlusion of the right main pulmonary artery either invaded or compressed by the mass, and no significant opacification of the right pulmonary arteries. The mass was further found to compress the superior vena cava with mild deviation to the left, also extending to the perihilar right upper lobe. A small right-sided pleural effusion was seen as well as a pericardial effusion measuring 1 cm anteriorly. A 1.2 × 0.5 cm pleural based nodule was found in the posterior left lower lobe. The patient was subsequently admitted to the hospital for further evaluation and a request for documentation of previous testing, including the “neurologic testing,” was placed (Figure 3).

**Figure 4:**
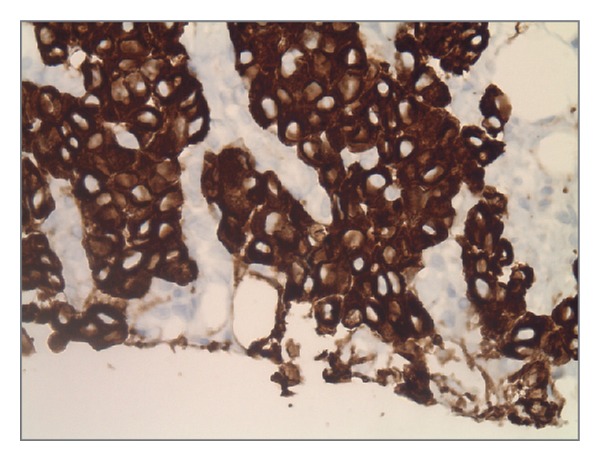
Biopsy of mass showing strongly CK7 positive.

**Figure 5:**
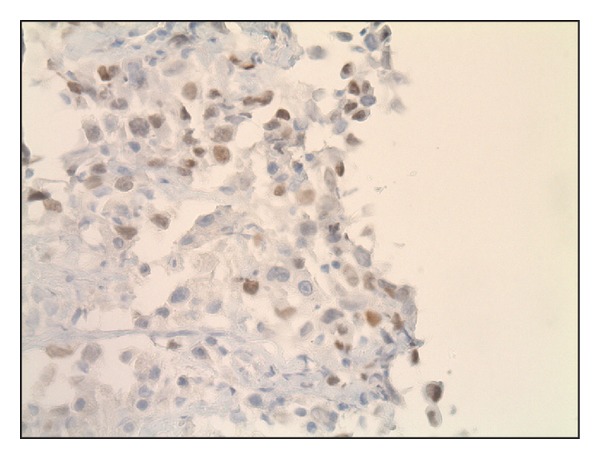
Biopsy of mass showing TTF-1 weakly positive.

On hospital day two, the patient was taken for CT guided biopsy of the mediastinal mass. Pathologic analysis of the sample was read as adenocarcinoma of the lung, poorly differentiated. Immunohistochemical stains showed positive for CK7 (Figure 4) and TTF1 (Figure 5) and negative for P63 and Napsin A markers.

Subsequently cardiothoracic surgery was involved to place a pericardial window for drainage and characterization of the pericardial fluid. Perioperative assessment revealed a hemodynamically stable patient. The patient denied any pain only mild fatigue and chest discomfort. Review of systems revealed remained similar to admission with a substernal dragging sensation and generalized feeling of fatigue. The neck range of motion remained slightly limited due to mild pain with flexion. A Mallampati grade 2 score was given to the individual with a thyromental distance of 8 cm, and sternomental distance of 10 cm, with natural dentition and no loose, chipped, or missing teeth. The patient complained of mild shortness of breath. The patient was subsequently intubated and received generalized anesthesia. Cytological examination of fluid obtained from the pleural effusion and pericardial fluid demonstrated pleomorphic malignant epithelial cells arranged in small clusters and glands and singly. Postsurgery recovery was complicated by inability to become extubated. The patient was subsequently placed in the intensive care unit. Due to prolonged intubation and inability to extubate the patient, it was then decided to place a tracheostomy tube for the patient.

On day ten of admission records of the nerve conduction studies completed upon initial presentation in the Dominican Republic demonstrated a decrease in the axillary nerve conduction velocity. Repeated nerve stimulation test showed a progressive decline in the compound muscle action potential (CMAP) amplitudes by 18.9%, and a more marked postexercise decrement in CMAP amplitudes was noted. These findings were reported as consistent with myasthenia gravis.

With discussion of the patient's prognosis and the patient's wishes with family members, a do not resuscitate order (DNR) was elected. On day 27 of admission, the patient was found to be in asystole and subsequently expired. A postmortem analysis was unfortunately not done.

## 3. Discussion

Pathological biopsy of the mediastinal mass in this patient confirmed the diagnosis of a centrally located adenocarcinoma of the lung. Cytologic reports of aspirated pleural fluid derived from the pleural effusions further correlated with this diagnosis. Reports of electrophysiologic testing completed one year prior to the current presentation were obtained and proved consistent with a diagnosis of myasthenia gravis. Serologic studies, however, were not completed on this admission to correlate with these findings and pose a limitation to the definitive diagnosis. Given the neurophysiologic diagnosis and sudden decompensation of the patient, a discussion of a possible stress induced myasthenic exacerbation after placement of the pericardial window is warranted.

Lung cancer is the most commonly diagnosed cancer worldwide accounting for 14% of the global cancer burden. In the United States, it has been the leading cause of mortality among both men and women [1]. In general, lung cancers are classified as two major groups: small cell lung cancer (SCLC) and non-small cell lung cancer (NSCLC) [2]. Of these, NSCLCs account for 85% of all lung cancers. The non-small cell lung cancer group is further subdivided to include adenocarcinoma, large cell carcinoma, bronchoalveolar carcinoma, and squamous cell carcinoma [3]. Adenocarcinoma of the lung constitutes the majority of NSCLCs, accounting for 40% of lung cancers. They are most commonly found in the periphery of the lung and associated with prior lung scars. Adenocarcinomas have the highest prevalence in previous nonsmokers and women more than men. At the time of diagnosis 20% are found as a localized disease, 25% with regional metastases, and 55% with widespread disease conferring the worst prognosis [2, 3].

Paraneoplastic syndromes are common findings in lung cancer. They often are incidental findings serving as a harbinger to the underlying disease. They comprise syndromes involving the neuromuscular junction, vascular, hematologic, and metabolic syndromes as well as connective tissue and skeletal tissue disorders [4]. Paraneoplastic disorders are diagnosed in up to 15% of patients with the highest incidence occurring in NSCLC. Neurological paraneoplastic syndromes have an incidence of 0.01% of cancer patients, often imposing a burden on the patient's ability to carry out their activities of daily living [5]. These neurological syndromes comprise syndromes such as Lambert-Eaton myasthenic syndrome (LEMS), limbic encephalopathy, polyneuropathy, cerebellar degeneration, opsoclonus-myoclonus, and autonomic neuropathy [4, 5].

Traditionally myasthenia gravis has been associated with several autoimmune diseases including lupus erythematosus, rheumatoid arthritis, diabetes mellitus type 1, hashimoto's thyroiditis, grave's disease, and part of the paraneoplastic spectrum in thymic diseases such as thymoma [6]. The correlation between myasthenia gravis and primary lung carcinomas has not been established, as has clearly been defined for Lambert-Eaton syndrome and small cell carcinoma of the lung. The association has often been regarded as coincidental [6, 7].

Antiacetylcholine receptor antibodies (Anti-AChR abs) are the causative agents in myasthenia gravis [6, 7]. These antibodies affect neuromuscular transmission by functional impairment of the AChR and accelerated degradation and complement activation at the AChR, ultimately leading to the loss of neurotransmission. 20% of these individuals, however, are referred to as seronegative patients. These individuals do not possess the classic anti-AChR abs rather they possess Anti-MuSK abs, an antibody towards argin/MuSK signaling pathway responsible for the functional maintenance of the postsynaptic neuromuscular junction [8].

The standard diagnosis of myasthenia gravis relies on the reliable demonstration of anti-AChR abs or anti-MuSK abs; however, these may not always be available. Electrophysiologic testing through repetitive nerve stimulation may be used alternatively to detect a neuromuscular transmission defect (sensitivity 95–99%).

The autoantibodies, anti-AChR or anti-MuSK, specifically target nicotinic acetyl choline receptors (nAChR). These receptors include the *α*1, *α*3, and *α*7 subunits, of which the *α*3 subunits are found within the thymus and postulated to induce myasthenia gravis [9]. In 1992, Chini et al. demonstrated the potential for small cell lung carcinoma, non-small lung carcinoma, and neuroblastomas to express the *α*3 subunit nAChR [10]. They further established the cross reactivity of autoantibodies against *α*3-nAChR against the a1 nAChR [10–12].

Patients diagnosed with myasthenia gravis have been known to present challenges to generalized anesthetic therapies. Due to the neuromuscular defect caused by this disease, patients are at an increased risk of pulmonary aspiration and overall difficulty of handling secretions [13, 14]. Factors influencing anesthetic risk include respiratory function which should be assessed with FVC measurements before surgery to assess the need for postintubation mechanical ventilation. Depolarizing agent resistance has been well documented in these patients with increased sensitivity to nondepolarizing agents [13, 14]. A shorter time of onset and longer duration of effects are generally experienced with nondepolarizing agents. These effects could be enhanced in patients receiving prior anticholinesterase therapy. Further volatile anesthetics, which enhance the impact of nondepolarizing agents, must be avoided or minimized. Overall agents such as barbiturates, propofol, etomidate, and ketamine have been used without event.

At the time of therapy with general anesthesia and a suspected diagnosis of myasthenia gravis, a more thorough examination of the cardiovascular and pulmonary systems was needed as well as serologic investigation for the diagnosis of myasthenia. Pulmonary function testing was not completed in the preoperative assessment of our patient. These studies may have allowed for prediction of poor postoperative and postgeneral anesthesia outcomes. Perhaps the rapid movement from CT guided biopsy to placement of the pericardial window could have avoided the myasthenic crisis experienced by this patient.

A myasthenic crisis may have been avoided with treatment modalities targeting a reduction of the tumor burden as well as immune modulation of the myasthenia gravis. The use of IV immunoglobulin (IVIG) and plasmapheresis (plasma exchange) may have given the best modality. IVIG is thought to protect against the damage caused by the autoimmune antibodies through neutralization, increase effector T-cells to maintain self-tolerance, and act as a distraction device to the host effector cells by interacting with their surface Fc receptors [15, 16]. Studies by Keime-Guibert et al. and Uchuya et al. [17, 18] studied the effects of using IVIG alone or IVIG plus cyclophosphamide and methylprednisolone. They showed that only 35–40% of patients achieved neurologic stability, with only one patient showing definitive improvement. The authors concluded that in patients with severe disability, this was an ineffective treatment modality.

Given these findings, one could expect the potential for development of myasthenia gravis-like symptoms in patients with SCLC, NSCLC, and neuroblastomas. Further, if so bold, it may warrant placement of myasthenia gravis-like syndrome on the list of paraneoplastic conditions associated with these conditions.

## Figures and Tables

**(a):** 

WBC (K/uL)	19.1
Hgb (m/uL)	15.6
HCT (%)	47.5
MCV (FL)	91.5
MCH (pg)	30.0
MCHC (g/dL)	32.8
RDW (%)	13.6
Platelet count (k/uL)	446

**(b):** 

Sodium (mmol/L)	137
Potassium (mmol/L)	4.0
Chloride (mmol/L)	96
CO_2_ (mmol/L)	29
BUN (mg/dL)	19.0
Creatinine (mg/dL)	1.0
Glucose (mg/dL)	106

**(c):** 

AST (IU/L)	20
ALT (IU/L)	48
Alkaline phosphatase (IU/L)	99
Total protein (g/dL)	7.2
Albumin (g/dL)	30.0
Bilirubin total (mg/dL)	32.8
